# Supplementary material for: Epigenetic gene regulation is controlled by distinct regulatory complexes utilizing specialized paralogs of TELOMERE REPEAT BINDING FACTORS
Source: PLoS Genet. 2026 Apr 21;22(4):e1012114. doi: 10.1371/journal.pgen.1012114 (PMC13132431; doi:10.1371/journal.pgen.1012114)
Supplement: S1 Fig — (PDF) [file pgen.1012114.s001.pdf]

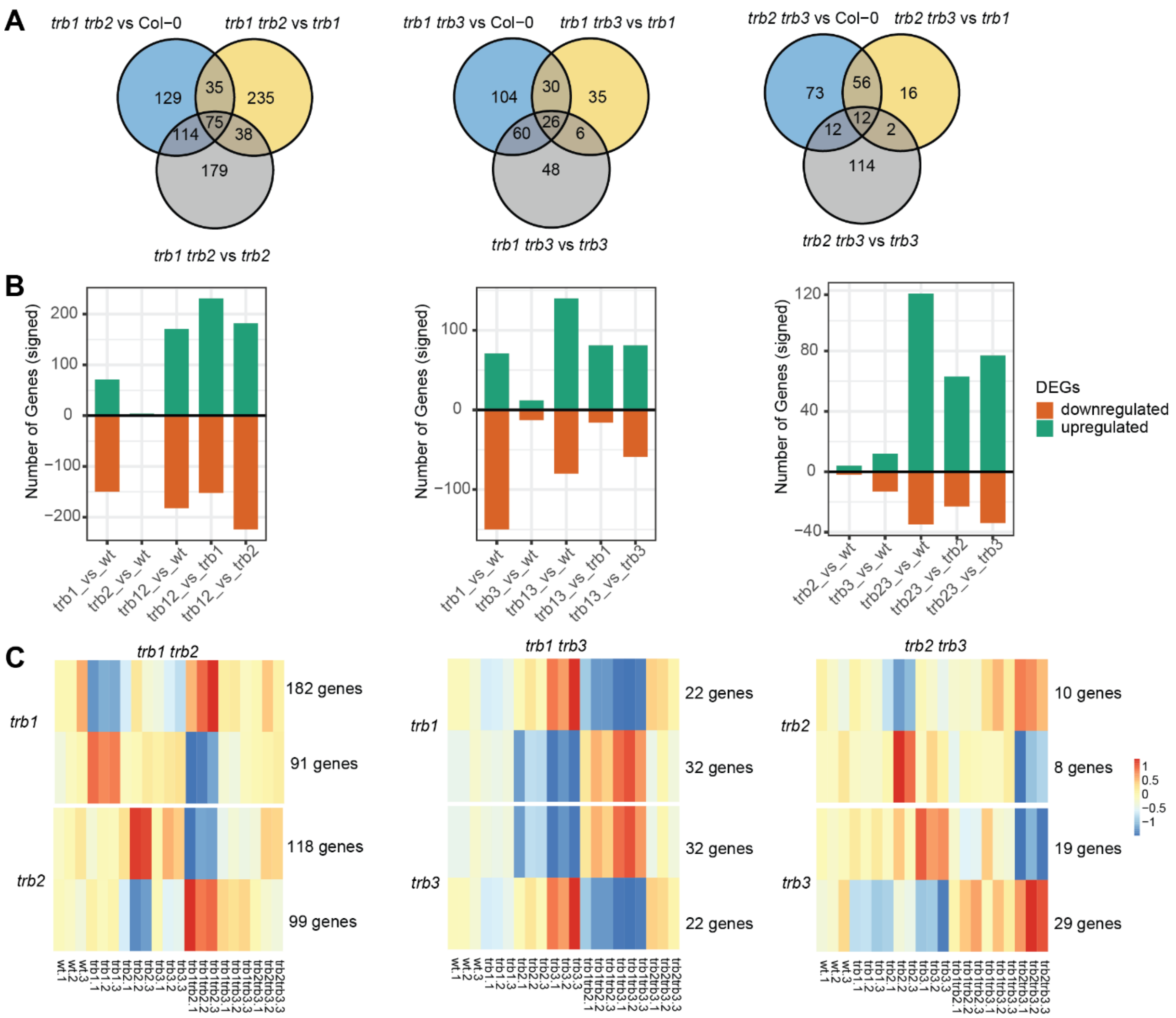

S1 Fig. DEGs detected between double and single mutants. A) Number of DEGs ($p<0.05$) in *trb1 trb2* versus *trb1* and *trb2* mutants (left), *trb1 trb3* versus *trb1* and *trb3* (middle) and *trb2 trb3* versus *trb2* and *trb3* (right). **B)** Direction of differential gene expression in comparison trb1 and trb2 single and double mutant (left), trb1 trb3 single and double mutants and trb2 and trb3 single and double mutant comparisons (right). Up and down genes are indicated by green and orange bars, respectively. C) Average of row normalized vst counts for 2 PAM clusters per double to single mutant comparisons as indicated on the top and left side of each subpanel, the number of genes in each cluster is indicated at the right.
