# Supplementary material for: Epigenetic gene regulation is controlled by distinct regulatory complexes utilizing specialized paralogs of TELOMERE REPEAT BINDING FACTORS
Source: PLoS Genet. 2026 Apr 21;22(4):e1012114. doi: 10.1371/journal.pgen.1012114 (PMC13132431; doi:10.1371/journal.pgen.1012114)
Supplement: S2 Fig — (PDF) [file pgen.1012114.s002.pdf]

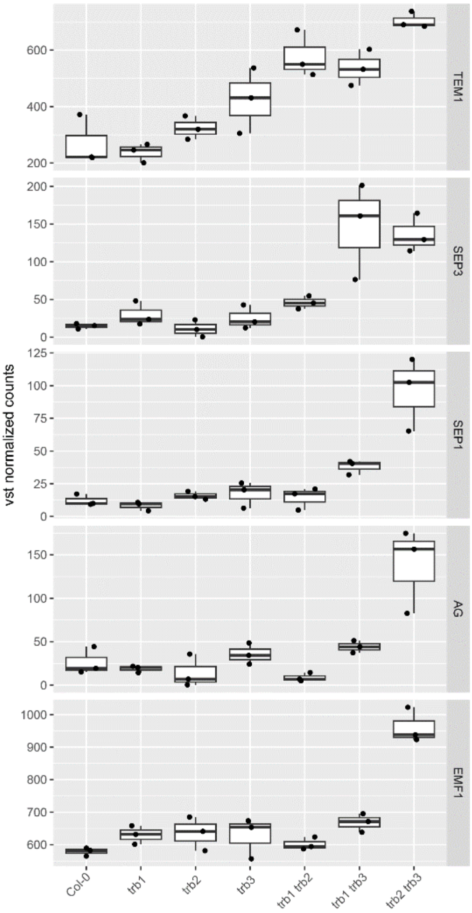

S2 Fig. Expression of flowering time pathway genes in *trb* mutants. Boxplots show variance stabilized read counts of RNA-seq analysis (n= 3 biological and experimental replicates) in *trb* single and double mutants. All genes were part of transcriptional cluster 4 as described in Figure 1. Boxplots show innerquartile range and median as line, each data indicated as dot.
