# Supplementary material for: Epigenetic gene regulation is controlled by distinct regulatory complexes utilizing specialized paralogs of TELOMERE REPEAT BINDING FACTORS
Source: PLoS Genet. 2026 Apr 21;22(4):e1012114. doi: 10.1371/journal.pgen.1012114 (PMC13132431; doi:10.1371/journal.pgen.1012114)
Supplement: S3 Fig — (PDF) [file pgen.1012114.s003.pdf]

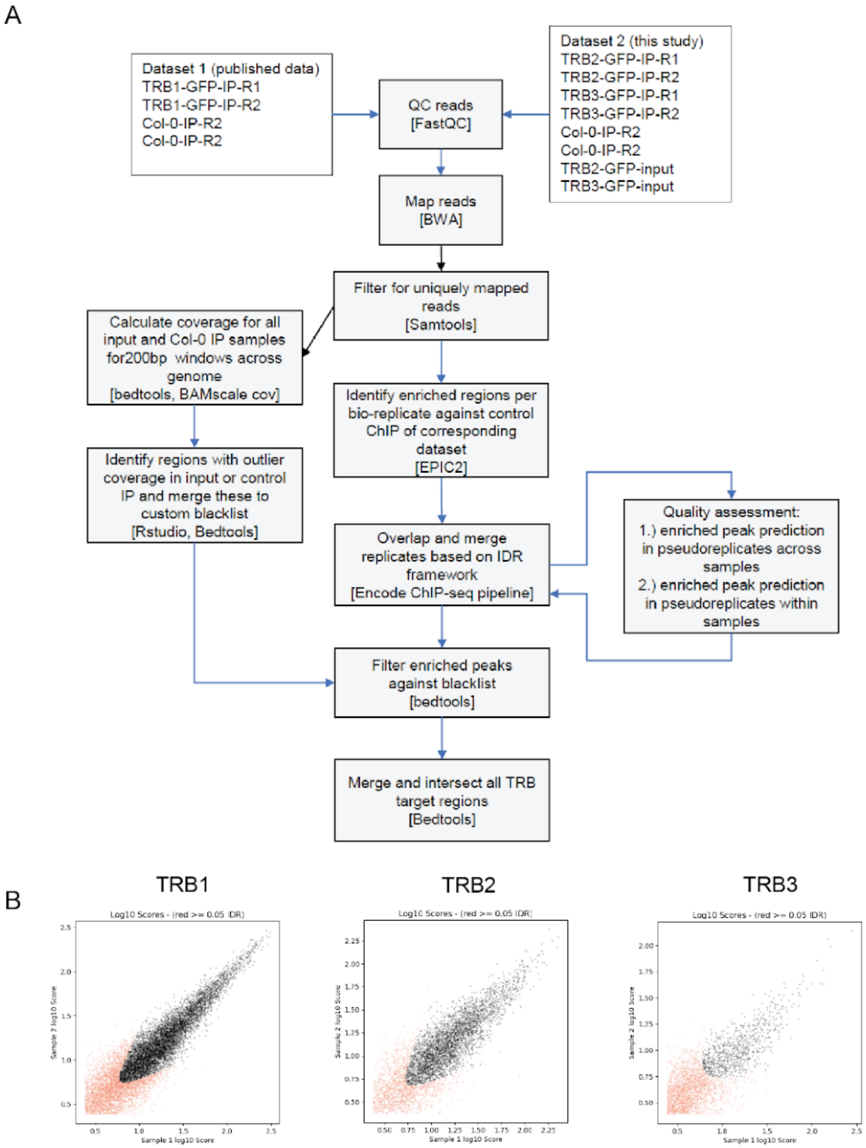

C

TRB1	TRB2	TRB3	IDR type
7483	3771	845	true replicates
8690	4321	1450	pseudo-replicates across samples
6607	3064	1155	pseudo-replicates within replicate 1
5545	3119	613	pseudo-replicates within replicate 2
1.2 (pass)	1.1 (pass)	1.7 (pass)	Np/Nt
1.2 (pass)	1.0 (pass)	1.9 (pass)	N1/N2

S3 Fig. Bioinformatics pipeline for ChIP-seq analysis and ChIP-seq quality control. A) Flowchart of the pipeline used for identification of enriched regions by ChIP-seq. **B)** Comparison of sample scores between 2 replicates (biological and experimental) using the Irreproducible Discovery Rate (IDR) framework as implemented by Encode. Samples were ranked by score determined by EPIC2, values with an IDR < 0.05 were considered as positive) **C)** Number of peaks identified by the pipeline in true replicates, pseudo-replicates and within sample replicates as indicated by IDRtype.
