# Supplementary material for: Epigenetic gene regulation is controlled by distinct regulatory complexes utilizing specialized paralogs of TELOMERE REPEAT BINDING FACTORS
Source: PLoS Genet. 2026 Apr 21;22(4):e1012114. doi: 10.1371/journal.pgen.1012114 (PMC13132431; doi:10.1371/journal.pgen.1012114)
Supplement: S4 Fig — (PDF) [file pgen.1012114.s004.pdf]

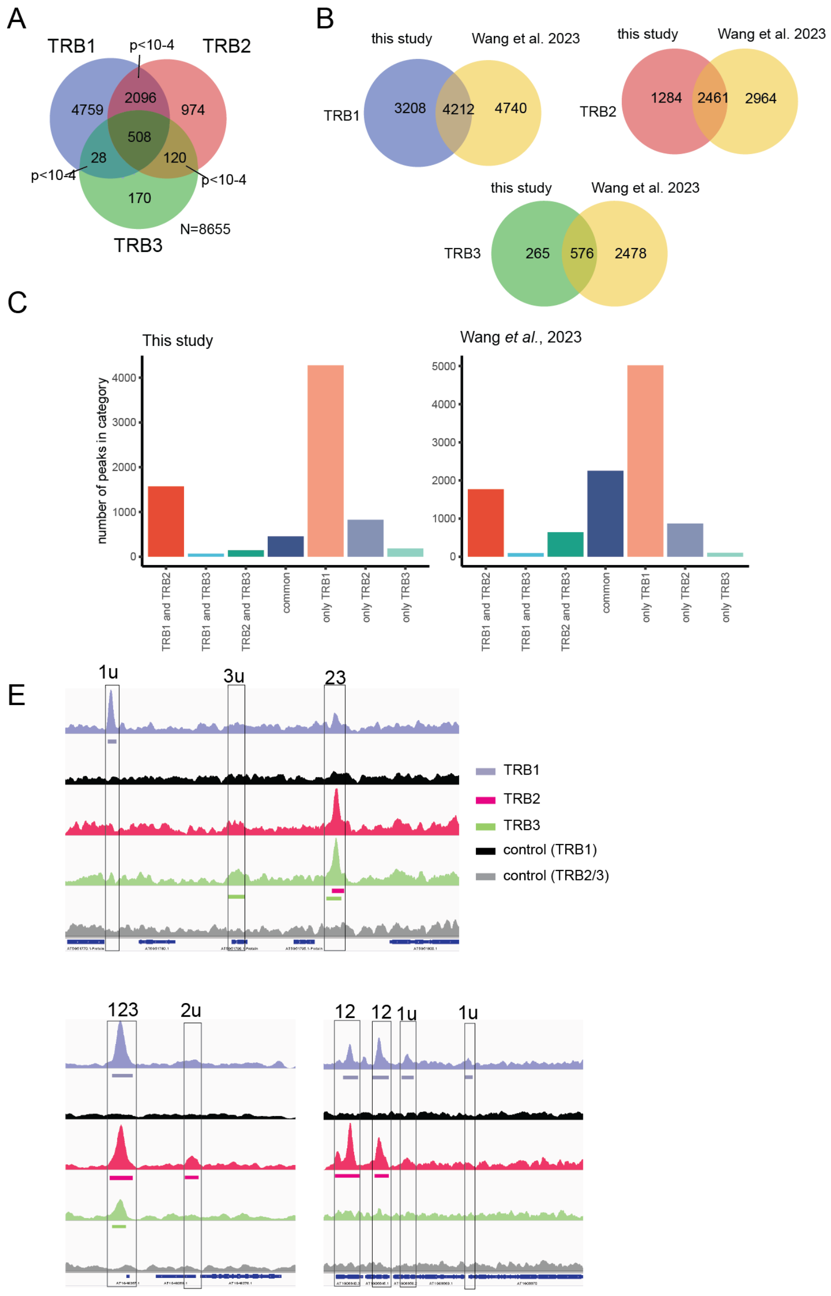

S4 Fig. Characterization of TRB1-3 ChIP-seq data. A) Venn diagram of peaks for TRB1-3. Peaks were back-annotated after merging all peaks to a common target site set. Statistical testing by permutation as implemented in Genome Association Tester (GAT). **B)** Venn diagram of peak overlap for TRB1, TRB2 and TRB3 between the current study and a previous study by Wang et al. 2023. **C)** Comparison of TRB1-3 peak categories identified in this study (left panel) and in the previous study by Wang et al. 2023. **D)** Genome browser tracks showing typical peaks (1u, 2u, 3u: only detected in TRB1, TRB2, TRB3. 12, 23: Peaks detected for TRB1 and TRB2 or TRB2 and TRB3. 123: peaks commonly detected). Background (control) shows ChIP-seq libraries prepared from Col-0. All tracks are normalized to Counts per million mapped fragments (CPM).
