# Supplementary material for: Epigenetic gene regulation is controlled by distinct regulatory complexes utilizing specialized paralogs of TELOMERE REPEAT BINDING FACTORS
Source: PLoS Genet. 2026 Apr 21;22(4):e1012114. doi: 10.1371/journal.pgen.1012114 (PMC13132431; doi:10.1371/journal.pgen.1012114)
Supplement: S5 Fig — (PDF) [file pgen.1012114.s005.pdf]

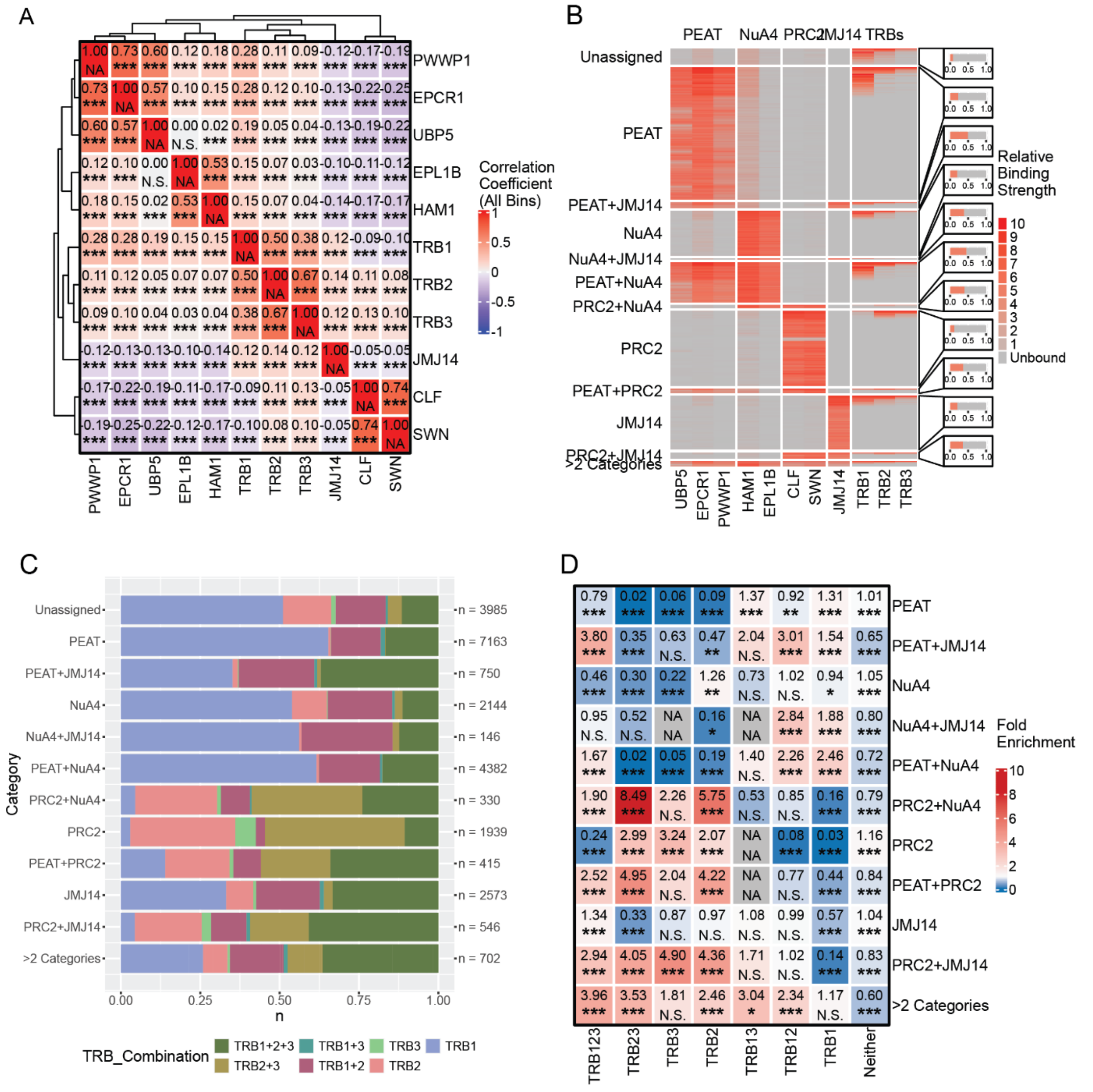

S5 Fig. TRB target and interaction partner correlation analysis using an independent dataset for TRB1, TRB2 and TRB3 A); Pairwise Pearson correlation matrix of ChIP-Seq peaks derived from eleven regulatory proteins and assigned to 200bp genomic bins. Significance levels: ***, p<=0.0005, **, p<=0.005, p<=0.05, N.S., p>0.05. **B)**; Left, Heatmap depicting the genomic bins bound by each of the ChIP-Seq sets used in A. Relative binding strength of each peak expressed through deciles. Columns depict proteins grouped by regulatory complex and rows depict bins assigned to complexes based on presence of at least two complex components. Bins assigned to more than two complexes were grouped into one category. *, bins which were neither assigned to complexes nor TRB-bound were excluded for the sake of readability. Right, Percentage of TRB-bound bins for each category. **C)** Distribution of TRBs in the TRB-bound bins of each category assigned in B. **D)** Pairwise exact test statistics for bins assigned to complex categories and their corresponding TRB combinations. Significance levels: ***, p<=0.0001, **, p<=0.001, p<=0.01, N.S., p>0.01.
