# Supplementary material for: Epigenetic gene regulation is controlled by distinct regulatory complexes utilizing specialized paralogs of TELOMERE REPEAT BINDING FACTORS
Source: PLoS Genet. 2026 Apr 21;22(4):e1012114. doi: 10.1371/journal.pgen.1012114 (PMC13132431; doi:10.1371/journal.pgen.1012114)
Supplement: S6 Fig — (PDF) [file pgen.1012114.s006.pdf]

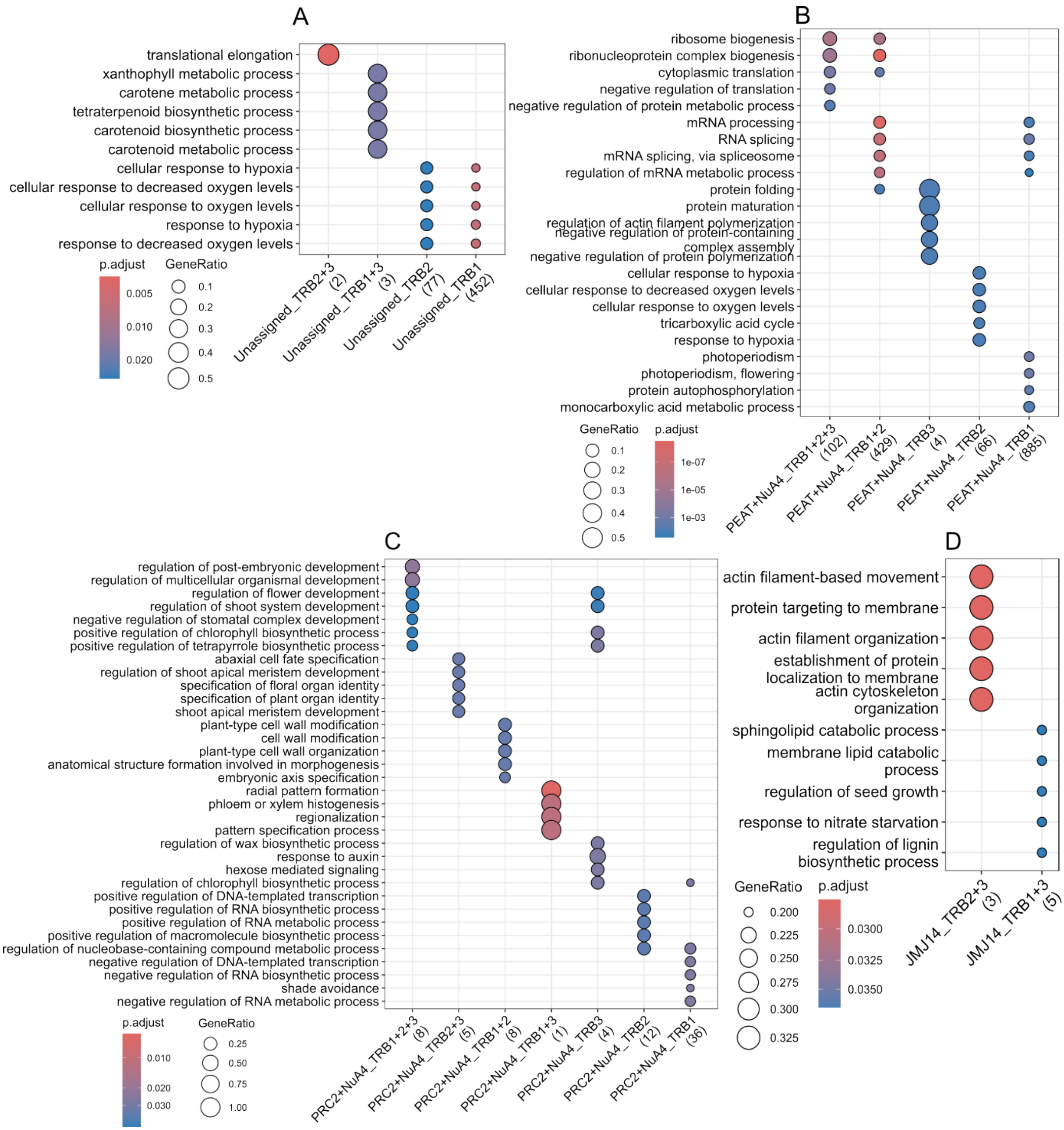

S6 Fig. Gene Ontology enrichment analysis. A) Enriched GO-Terms for the un-assigned gene set separated by TRB paralog. **B**) Enriched GO-Terms for the gene set assigned to PEAT and NuA4 complexes separated by TRB paralog. **C**) Enriched GO-Terms for the gene set assigned to PRC2 and NuA4 complexes separated by TRB paralog. (**D**). Enriched GO-Terms for the gene set assigned to JMJ14 separated by TRB paralog. The 5 most enriched "Biological Process" terms per combination were selected and similar terms reduced into one representative term. Full list of enriched GO-terms available in Supplemental File 5.
