# Supplementary material for: Epigenetic gene regulation is controlled by distinct regulatory complexes utilizing specialized paralogs of TELOMERE REPEAT BINDING FACTORS
Source: PLoS Genet. 2026 Apr 21;22(4):e1012114. doi: 10.1371/journal.pgen.1012114 (PMC13132431; doi:10.1371/journal.pgen.1012114)
Supplement: Attachment — Submitted filename: Response_to_Reviewers_version2.docx [file pgen.1012114.s012.docx]

**Response to reviewer’s comments**

We would like to thank all reviewer for the general support and the detailed comments. The reviewers suggested additional analyses. We feel that the they helped us to improve our manuscript considerably and made the conclusions much more robust.

We provide a document with changes underlined in yellow as additional file. Please find our point-by-point replies below.

**Comments to the Authors:
Please note here if the review is uploaded as an attachment.**

## Reviewer #1:

Three TRB proteins have been identified as shared components of several chromatin-associated complexes, including PEAT, PRC2, and NAC-JMJ14. These TRB proteins were previously thought to function redundantly within these complexes to regulate chromatin status and transcription. Interestingly, this study reveals non-redundant roles for the TRB proteins, providing important insights into how they exert specialized functions in chromatin regulation and transcriptional control. In this study, integrated analyses based on genetic, RNA-seq, and ChIP-seq data were performed. The main conclusions are well supported by the results presented in the manuscript. However, because the phenomena described are complex, the presentation of the results is somewhat difficult to follow. I suggest that the authors describe the results in a more concise and streamlined manner to improve clarity and accessibility for a broader readership. Additional comments are provided below:

Major points:
1. It is interesting to find that the prope triple mutants that segregated functional alleles of TRB2 showed a significant early-flowering phenotype. Because TRB proteins have been shown to form a protein complex with JMJ14 and NAC050/052, this observation is consistent with prior reports describing early-flowering phenotypes in nac050/052 and jmj14 mutants. This needs to be indicated in the manuscript.

At the moment, we cannot say why exactly the *trb1 trb3 trb2+/-* *prope* triple mutant is slightly early flowering. Transcriptional cluster 5 (in the revised version) enriches the Wikipath flowering and is linked to gene repression, but in this cluster TRB2 and TRB3 seem to have redundant roles.

Concerning the link to JMJ14/NAC50/52, the causal gene(s) for early flowering observed in *jm14* and *nac50/52* is(are) also not clearly identified. Jeong et al (PLOS One, 2009) reported a direct effect on *FT* expression, an observation that was refuted by Lu et al. (Cell Research 2010). Ning et al (NAR, 2015) found increased expression of *FT* in *nac50/52*, *jmj14* and combined mutants, but unfortunately looked at plants that had already transitioned to flowering. In this manuscript, a cross of *jmj14* to *ft* mutants showed unambiguously that early flowering was not only dependent on *FT* nor was it fully dependent on FVE, a gene in the autonomous pathway.

We have added a short paragraph on flowering to the discussion (including the reference to Ning et al.), but we do not want to speculate too much. It is a point for which future studies are required.

2. In line 182, the text indicates that "Obvious positive correlation was also observed between PEAT, NuA4, TRB1 and TRB2 and between TRB3 and PRC2". However, TRB3 shows a relatively high positive correlation with TRB-JMJ14 components, but shows low positive correlation levels with all other components. The correlation levels between TRB3 and PEAT components (PWWP1 and EPCR1) are even higher than the correlation between TRB3 and PRC2 components (CLF and SWN). The correlation between TRB3 and PRC2 requires cautious interpretation and should not be overstated.

In response to a point of reviewer 2, we have now re-run our analysis with smaller genomic bins that are able to split closely adjacent peaks. For PRC2, the new analysis fits better to our written statement: TRB3 bins are clearly positively correlated to bins of PRC2 catalytic subunits, TRB2 shows a weak positive correlation but TRB1 a negative correlation. For JMJ14, all TRBs are correlated with the order TRB2>TRB1>TRB3. For PEAT components all correlations with TRB bins are positive but the order is TRB1 >TRB2 >TRB3. Overall, TRB3’s scores are PRC2 > JMJ14 >PEAT.

3. The pairwise Pearson correlation matrix shown in Figure 2A is important for understanding the non-redundant functions of different TRB proteins. One confusing observation is that the ChIP-seq signals of PRC2 components (CLF and SWN) show only a weak negative correlation with those of the NuA4 component EPL1B. This analysis therefore appears insufficient to clearly distinguish repressive chromatin regions marked by PRC2 from active chromatin regions marked by NuA4. This issue should be resolved in the revised manuscript.

As above, the improved analysis with smaller bins shows clear negative correlations between PRC2 and PEAT or NuA4 components.

4. The DNA-binding affinities of TRB1 and TRB3 to different DNA fragments were compared, showing that the overall binding affinity of TRB1 was lower than that of TRB3, even for the R4 probe (Figure 4C). Notably, the purified TRB1 protein appears to be of lower quality than the TRB3 protein, as shown in Figure 4B. Therefore, the quality of the MST data should be improved to more convincingly confirm the differences in DNA-binding affinities between TRB1 and TRB3.

As the reviewer mentioned, we could never purify TRB1 protein from *E. coli* without signs of degradation or partial translation; furthermore, we could not concentrate the TRB1 protein to the same extend as TRB3 as it precipitated more easily. We do not agree that the affinity against the R4 telobox was significantly less for TRB1 as it was more or less identical (568 nM of TRB1 and 531 nM, respectively). The affinity corresponds to a prior measurement by Hofr et al. 2009. We found that encouraging and argued that despite the differences in protein quality, we could meaningfully compare affinities for other probes. Even if we slightly underestimate the affinity of TRB1 for single telo-box or CRACCTA motifs, it is clear that, alone, TRBs are not high affinity DNA-binding proteins. This is the main conclusion from our figure.

Minor points:
1. In line 65 and 66, "HAM1 and HAM2 which links PEAT to the NuA4 complex". This statement may cause misunderstanding, because PEAT and NuA4 complexes are independent complexes, and HAM1 and HAM2 are shared subunits of these complexes.

We have amended the text as follows:

“Histone acetylation is facilitated through HISTONE ACETYLASE RELATED TO MYST (HAM) 1 and 2 which are shared components of PEAT and the Nucleosomal Acetyltransferase of histone H4 (NuA4) complex[[11]](https://sciwheel.com/work/citation?ids=15523443&pre=&suf=&sa=0).”

2. Figure 4A contains words with too small font, and the resolution of the figure is low. Please increase the size and resolution.

We have improved this by reducing the “heat-map” field’s sizes.

3. In line 227, "Figure 4" should be changed to "Figure 4A". The results described in line 235- 248 should contain referenced figures.

We have added the precise reference to Figure 4A.

4. In line 271, "Figure 4G and G" should be changed to "Figure 4F and G".

Corrected

5. The resolution of images shown in Figure 4A and Figure 6C should be improved, and the front size should be increased.

Done as suggested, we now used a legend so that there are less in-figure letters but hopefully better clarity

6. In line 227, replace the citation "Figure 4" with "Figure 4A".

See above, done as suggested

7. In line 271, correct the citation "Figure 4G and G" to "Figure 4F and G".

See above, done as suggested

## Reviewer #2

In this manuscript, Maik Mendler and colleagues have attempted to address the redundancy between three telomere repeat binding transcription factors in target loci selectin and protein interactions. Redundancy among family members is a long-standing obstacle in understanding the function of each member. By using traditional forward and reverse genetic methods, the diversity among family genes is usually not be fully addressed due to the functional redundancy. In this work, the authors incorporated multiple genome-wide data and various bioinformatic methods to dissect the function of three TRB TFs, the DEG, the target selection, the associated regulatory protein complexes, and most importantly, the distinct biological function. The paper is well written, with rational and line of reasoning. The work is genuinely novel, orginal, and would provide insightful methodology of the field.

Specific Comments:
In the first part of the result section, the RNA-seq was conducted in single, double and triple mutants of trbs, and performed PAM clustering to assemble the genes into 4 groups. It would be better if DEGs were separated into a up and down genes first to give a direct view to the role of TRB in gene regulation.

We have done the analysis and confirm that the results are more informative. We have added a new panel to Figure 1 with the result of the analysis (Figure 1D). In the case of single mutants, only *trb1* shows a significant number of differentially expressed genes in the comparison to wt, with a majority showing decreased over increased expression. Double *trb1 trb2* mutants are more or less balanced between up- and down DEGs. In contrast, trb1 trb3 and trb3 trb2 show more DEGs with increased than decreased expression, although in numbers, they have less upregulated DEGs than *trb1 trb2*.

In target gene part, the TRB1 targets were derived from published dataset, which showed a greater number of targets compared to TRB2 and TRB3. How could the authors prove that this difference is due to the character of the TFs but not the different conditions in generating the data.

Supplemental Fig 3B showed the comparison of number of peaks, but not the target, which is not effective for the comparison between their data and published data. In addition, it is better to give the general metaplot of the thee TRBs first, to give a comprehensive view of the genome-wide binding.

The reviewer raises very critical points, our answer addresses these together.

For all datasets from our group, the same growth conditions and ChIP protocols were used. The material was collected at ZT14 from 14-day-old seedlings tissue-culture seedlings grown in LD, as caveat, TRB2 and TRB3 were expressed under their native promoter while TRB3 was expressed under the control of the 35Sp promoter. There are two questions: first does the level of expression change the number of targets, second, does the level of expression change the nature of targets.

To provide an answer, we also looked at the second published dataset that reported on genomic fusion of TRB1,2,3 to FLAG epitopes [(Wang et al. 2025)](https://sciwheel.com/work/citation?ids=18325327&pre=&suf=&sa=0&dbf=0). These ChIPseq data used different growth and experimental conditions and a different tag.

Comparing our data set with the published data from the Jacobsen Group we see more targets in the latter, but a similar distribution with TRB1> TRB2>TRB3. We had already included this figure in the submitted manuscript, it has now become Supplemental Figure 4C). The difference between TRB2 and TRB3 is larger in our set than in the Jacobsen set while the large number of TRB1 targets is quire similar in both sets.

Since these differences and only partial overlap between the sets is somewhat disconcerting (see new panel B in Supplemental Figure 4), we performed the entire correlation analysis between TRB targets and interacting partners independently for both, our and the dataset from Wang et al. This analysis (New supplemental Figure 5) revealed that despite the differences in the number of peaks, the correlations between TRBs and other complexes hold true, indicating that the nature of peaks is quite similar. The only differences were detected between Figure 2C and Supplemental Figure 4C, where the TRB1 + TRB2 + TRB3 categories is larger while the category TRB2 + TRB3 and TRB1 only is relatively reduced (it is still best visualized in Supplemental Figure 4C). This shift could be explained by relatively more TRB3 targets in the set from Wang et al..

Combining both analyses, we are now more confident than before that the general correlation between TRBs and binding partners are robust; furthermore, there is a clear indication of preferred association single TRBs and different chromatin complexes. We debated whether we should also look at the “core set” of TRB targets identified in both studies or the union of all identified peaks. In the end, the better analysis would be to perform new ChIP experiment with the same protocols (and lines) but in varying growth conditions since this would reveal whether the participation of TRBs in their associated complexes is dynamic. It is beyond the scope of a revision, but an interesting point that would not have become so obvious without the reviewer’s suggestion. We have added the point to the discussion.

Line 177, the bin was 500 bp in the analysis. However, since the authors tried to associate TRBs with histone modifiers, the 500 bp bin is roughly too large, which spanned more than three nucleosomes. The authors could consider using a narrow bin within two nucleosomes for the correlation analysis.

After following the reviewer’s suggestion, we found indeed a sharper separation between predominantly activating and repressing signatures in the Pearson’s correlation and a better differentiation between TRBs (see also reply to reviewer 1 on how TRBs correlate to different complexes). We replaced the previous figure panels with the analysis performed with 300bp windows. 300bp correspond to 1-2 nucleosomes and the fragment length of our TRB ChIP experiments.

P7 line 206, the IP-MS conducted with TRB1 and TRB3 and TRB2 was missing. Moreover, the genes were overexpressed, but TRB1 and TRB3 were quantitively compared. The over amount of TRB proteins may obscure the quantitative comparative analysis of their interactors.

See above, we agree that the amount of protein may result in differential interactions either with DNA or with other proteins. In this case, however, both lines expressed TRBs under the control of the 35S promoter. Any bias was common to both lines and differential enrichment was the exception not the rule. We argue that since most interactors were not differentially enriched the bias detected for 13 out of 96 co-purified proteins is meaningful. The bias that we observed fits into the final conclusion that TRB1 is more likely to act within PEAT while TRB3 is more likely to act together with PRC2, while there may be less bias for JMJ14 and ICU11 interactions.

For the MST analysis, TRB2 was missed.

The point of the MST analysis was to find out if TRB1 had a preference for non-canonical telo-box like motifs that were specifically enriched only for TRB1. This seems not to be the case. Adding TRB2 to the analysis would not improve the conclusion on this point.

Lastly, the biological function and targets of each TRB1 with their distinct regulatory complex were assigned. It was revealed that a specific TRB sites could be associated with several complexes. What experimental strategies could the authors adopt to validate the bioinformatic analysis? This is quite interesting, since the conclusion were based on multiple bioinformatic methods and co-operation of a single locus are usually observed for plants to tackle with various environmental stimuli and internal signal. Experimental validation will not only enhance the robustness of this work, but also shed light to the field.

We have added a few suggestions of future research directions to the discussion.

For example, considering the incomplete overlap of TRB targets from different groups and the absence of TRBs from many partner’s binding sites, future work should evaluate if TRB-complex associations are condition dependent. Some of these conditions could enhance ar revieal phenotypic effects.

For the flowering response, it would be interesting to identify the genes that are causal and why TRB2, which sees somewhat less selective in target preference than TRB1 and TRB3, is the “weak” allele.

Both points could relate to modified activity of single TRBs in different conditions, but it may also be the availability of complex partner.

We agree that a detailed study of single target genes that are co-bound by different complexes are very interesting and could give more insight, e.g. by using targeted mutagenesis to abolish binding sites.

Reviewer #3:

Mendler et al. provide comprehensive RNA-seq and ChIP-seq data to dissect the target gene selection among TRB1, TRB2, and TRB3. Comparing the target sites of TRB proteins with those of a set of epigenetic regulators suggests that TRB proteins may target genes by associating with distinct epigenetic complexes. For example, the binding sites of TRB1 and TRB3 are strongly associated with the PEAT complex and PRC2, respectively. The interacting proteins co-purified with TRB1 and TRB3 further support this notion, as the PEAT complex subunits EPCR1 and EPCR2 are preferentially enriched by TRB1, while EMF1 is exclusively enriched by TRB3. Thus, this manuscript provides a mechanism for TRBs' target gene selection through preferential association with distinct epigenetic regulators.
Here, I list my concerns about this manuscript below:
1. The authors compared the transcriptome data among TRB single mutants and double mutants. It would be more appropriate to compare single mutants with their respective double mutants, such as comparing trb1, trb2, and trb1 trb2. Given that only about one-third of the differentially expressed genes (DEGs) identified in single and double mutants are shared with trb123, the authors should discuss this point.

We performed the analysis suggested by the reviewer and found that a number of targets were specific to the comparison double mutant versus single mutant. We had not suspected that for trb3 and trb2 single mutants which showed no real differences to Col-0. The single mutant specific targets seem to be explained by non-signficant expression trends observed in the single mutant to Col-0 comparison that enhance to potential to detect significant change in double mutants if they are inversed. We performed expression pattern clustering to detect this trend. All analyses (including comparing up- and down-regulated genes as suggested by reviewer 1) were added as new Supplemental Figure 1.

For the main analysis of DEGs, we used the union of all DEGs (Heatmap in Figure 1F). The increased number of genes were best split in 5 clusters (previously 4). During the re-analysis, we also realized that *trb2.1* was an outlier to all other samples. We removed trb2.1 from the DEG and cluster analysis, but included it in the heatmap (see amended methods).

Concerning the comparison to *trb123*, it is true that the number of DEGs is much higher – corresponding to the strong developmental phenotype. In a way, this was the starting point of the manuscript – how to explain this high redundancy while at the same time observing a preference of single TRBs for certain complexes.

2. In line 115, these mutants should be specified as trb123.

We have now more explicitly names trb123 mutants in line 102 “Triple *trb1-2 trb2-3 trb3-2* (*trb123*) mutants”. In line 115, we have added “mutants” to trb123

3. The binding sites of TRB proteins far exceed the DEGs; therefore, the relationship between TRB-regulated genes and TRB binding sites should be discussed.

Absence of repression does not cause great changes in gene expression unless there are also activators. This has been shown many times, e.g. *clf swn* sporophytes have almost no H3K27me3, yet only a small proportion of genes is upregulated. Apparently the same holds true for activators, their absence does not necessarily repress genes unless a repressor is also present. We have added this point to the discussion.

4. The authors selected CLF and SWN binding sites as representatives of PRC2 targets [1]. However, these binding sites are significantly fewer than the total number of PRC2 target genes. Only a small fraction of genes exhibit reduced H3K27me3 in their mutants, making it inappropriate to select these datasets to represent PRC2 targets. Utilizing the FIE binding sites may provide a better alternative [2].

The rational of using CLF and SWN was that TRBs physically bind to these proteins; thus, common binding would potentially represent co-recruitment. CLF and SWN show high redundancy and the double mutant is depleted of H3K27me3 in the sporophyte. It is rather similar to what we observe for TRB’s redundancy, actually. Importantly, the ChIPseq quality of this published dataset is very good and we prefer it over the published FIE dataset, which was generated in the accession Ws.

5. In lines 265-266, the rationale for choosing the SEP3 promoter should be clearly presented.

In Zhou et al. 2028, we had established that the *SEP3* telo-box motifs is important for epigenetic repression of the locus. We have added this information and the reference to the description of the experiment.

1. Shu J, Chen C, Thapa RK, Bian S, Nguyen V, Yu K, et al. Genome-wide occupancy of histone H3K27 methyltransferases CURLY LEAF and SWINGER in Arabidopsis seedlings. Plant Direct. 2019;3(1):e00100. doi: 10.1002/pld3.100. PubMed PMID: 31245749; PubMed Central PMCID: PMCPMC6508855.
2. Zhou Y, Wang Y, Krause K, Yang T, Dongus JA, Zhang Y, et al. Telobox motifs recruit CLF/SWN-PRC2 for H3K27me3 deposition via TRB factors in Arabidopsis. Nature genetics. 2018;50(5):638-44. doi: 10.1038/s41588-018-0109-9. PubMed PMID: 29700471.
